# Hydrogen gas distribution in organs after inhalation: Real-time monitoring of tissue hydrogen concentration in rat

**DOI:** 10.1038/s41598-018-38180-4

**Published:** 2019-02-04

**Authors:** Ryo Yamamoto, Koichiro Homma, Sayuri Suzuki, Motoaki Sano, Junichi Sasaki

**Affiliations:** 1 Department of Emergency and Critical Care Medicine, Keio University School of Medicine, Tokyo, Japan; 2 Department of Cardiology, Keio University School of Medicine, Tokyo, Japan

## Abstract

Hydrogen has therapeutic and preventive effects against various diseases. Although animal and clinical studies have reported promising results, hydrogen distribution in organs after administration remains unclear. Herein, the sequential changes in hydrogen concentration in tissues over time were monitored using a highly sensitive glass microsensor and continuous inhalation of 3% hydrogen gas. The hydrogen concentration was measured in the brain, liver, kidney, mesentery fat and thigh muscle of rats. The maximum concentration, time to saturation, and other measurements representing the dynamics of distribution were obtained from the concentration curves, and the results obtained for different organs were compared. The time to saturation was significantly longer (20.2 vs 6.3–9.4 min. *P* = 0.004 in all cases) and increased more gradually in muscle than in the other organs. The maximum concentration was the highest in liver and the lowest in the kidney (29.0 ± 2.6 vs 18.0 ± 2.2 μmol/L; *P* = 0.03 in all cases). The concentration varied significantly depending on the organ (*P* = 0.03). These results provide the fundamentals for elucidating the mechanisms underlying the *in vivo* favourable effects of hydrogen gas in mammalian systems.

## Introduction

Hydrogen gas (molecular hydrogen) has emerged as an attractive medicinal agent due to its therapeutic and preventive effects in various diseases^1–7^. Several studies on animals have shown that the hydrogen reduces the reactive oxygen species (ROS) in tissues^1,4–6,8^ and has other antioxidant and/or anti-inflammatory effects on ischaemia-reperfusion injury in which the ROS are produced in excess^4,9,10^. Some clinical investigations have also shown the beneficial effects of inhaled hydrogen gas on acute myocardial infarction and out-of-hospital cardiac arrest^4,11^. Furthermore, studies regarding the safety and feasibility of hydrogen gas have reported that the hydrogen gas can be supplied to patients with a simple device that can be adapted to various clinical settings^11,12^.

Although hydrogen gas is produced by intestinal microbiota via fermentation of sugars and a considerable amount can be detected in breath^13^, the physiological mechanism by which the artificial administration of hydrogen provides favourable therapeutic effects remains unclear^14^. While recent studies have reported that the hydrogen gas affects cell signal transduction and may prevent cellular apoptosis^15,16^, another work reported that the oxidation-reduction reactions involving molecular hydrogen only occur with strong ROS, which cause tissue injury and not with weak, beneficial ROS^14^.

Despite such promising data and increasing popularity, studies investigating the hydrogen gas distribution or dynamics in organs after administration are generally lacking. Since hydrogen gas has low molecular weight and can rapidly diffuse through cell membranes^1,17^, it is believed that the concentration of hydrogen in the body would reach saturation shortly (minutes) after administration^9^. However, one study estimating the hydrogen concentrations in homogenised rat tissues revealed different peak concentrations among organs following one-time inhalation or intraperitoneal administration of hydrogen^18^. The same study also proposed variable dynamics of hydrogen concentration in organs over time^18^. In addition, there is a considerable debate on the administration method(s) that would provide hydrogen efficiently to the target organs^19^.

In an effort to clarify the hydrogen tissue distribution and dynamics after supply, the present study examines the sequential changes of hydrogen concentration in rat organs and tissues over time with continuous initiation of 3% hydrogen gas. The hydrogen concentration was measured in several organs by real-time monitoring with a highly sensitive glass microsensor, which is a practical method to measure hydrogen concentration *in vivo*.

## Results

### Definition of hydrogen concentration measurements

**Figure 1:**
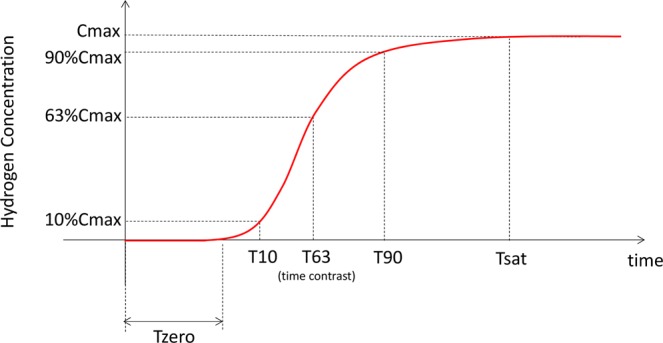
Definition of hydrogen concentration curve measurements. Important concentration curve measurements were as follows. C_max_ - the maximum plateau concentration where the concentration changes were less than 1% of the difference between baseline and maximum concentrations (i.e., saturation) over 1 min. C_max-adjusted_ - C_max_ values adjusted by microsensor signals obtained before staring the inhalation and used as an *in vivo* calibration. T_sat_ - the time between initiation of inhalation and beginning of saturation. T_10_, T_63_, and T_90_ - the time taken to reach 10, 63, and 90% of C_max-adjusted_, respectively. T_zero_ - the time required for the hydrogen concentration to reach 3% of C_max-adjusted_.

The values of concentration provided by the real-time monitoring were fit to a first-order lag curve. Then, some substantial measurements from concentration curves were selected based on the basics of physics (Fig. 1) and compared to elucidate the differences in hydrogen distribution in organs. C_max_ (i.e., saturation) was defined as the concentration on the highest plateau where changes in hydrogen concentration were less than 1% of the difference between baseline and maximum concentrations over 1 min. C_max_ was then adjusted using the microsensor signals obtained before hydrogen gas inhalation and used as an *in vivo* calibration (C_max-adjusted_). T_sat_ was defined as the time between the initiation of gas inhalation and beginning of the C_max_ plateau (saturation) while T_10_, T_63_, and T_90_ represent the time taken to reach 10, 63, and 90% of the C_max-adjusted_ value, respectively. T_63_ represents the time constant, a parameter that reliably characterises the speed of changes in time-invariant curves. T_zero_ was the duration of the initial (lowest) plateau, defined as the time needed for the hydrogen concentration to reach 3% of the C_max-adjusted_ value. T_zero_ was then adjusted to fit the concentration curves to a first-order lag model.

### Maximum Concentration (C_max_) for hydrogen

**Figure 2:**
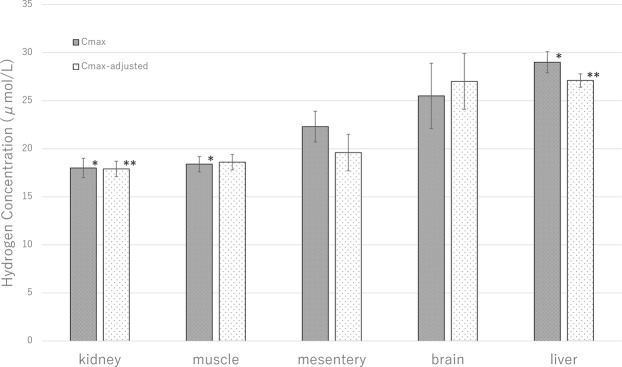
Maximum hydrogen concentration (C_max_). The liver had the highest mean C_max_ while the kidney had the lowest. Although the C_max_ varied significantly depending on the organ (*P* = 0.02 by analysis of variance), the inter-organ comparisons only revealed significant differences between the liver and kidney (*P < 0.05 by unpaired *t-*test) and liver and muscle (*P < 0.05 by unpaired *t-*test). C_max-adjusted_ was also the highest in the liver and the lowest in the kidney. C_max-adjusted_ was significantly different between the liver and kidney (**P = 0.04 by unpaired *t-*test).

C_max_ values for each organ are shown in Fig. 2. The highest mean C_max_ was found in the liver (29.0 ± 2.6 μmol/L) and the lowest in the kidney (18.0 ± 2.2 μmol/L). Although the C_max_ varied significantly depending on the organ (*P* = 0.02 by analysis of variance), the inter-organ comparisons only revealed significant differences between the liver and kidney (*P* = 0.03) and liver and muscle (18.4 ± 1.7 μmol/L in the muscle, *P* = 0.04). The distribution of C_max_ among the target organs remained the same after *in vivo* calibration (C_max-adjusted_). The C_max-adjusted_ value was also the highest in the liver (27.1 ± 1.8 μmol/L) and the lowest in the kidney (17.9 ± 1.9 μmol/L). The C_max-adjusted_ was significantly different between liver and kidney (*P* = 0.04).

### Hydrogen distribution and saturation dynamics

**Figure 3:**
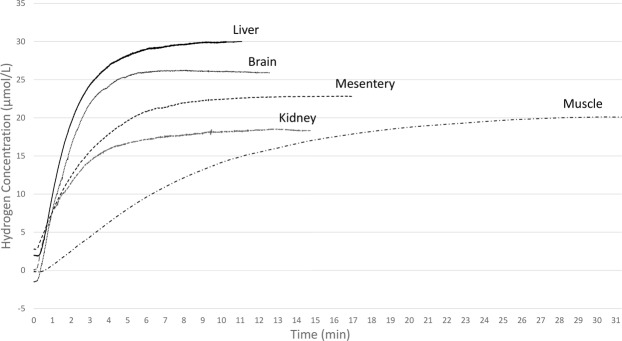
Hydrogen distribution curve until saturation. The hydrogen concentration increased more gradually in thigh muscle compared to the other organs. The liver had the highest C_max_ while the kidney had the lowest. Liver, *n* = 6, brain, *n* = 8, mesentery fat, *n* = 4, kidney, *n* = 5, thigh muscle, *n* = 5.

**Figure 4:**
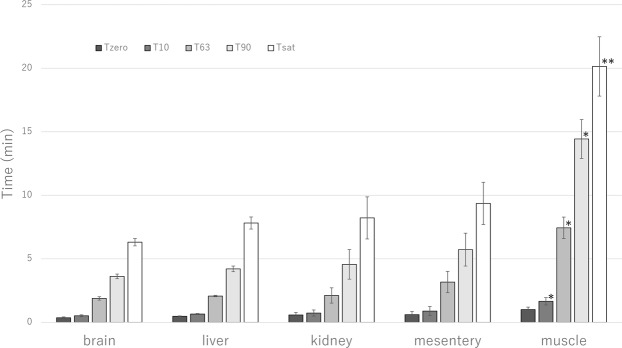
Hydrogen saturation dynamics. Time to saturation (T_sat_) and the time taken to reach 10% (T_10_), 63% (T_63_), and 90% (T_90_) of adjusted C_max-adjusted_ were all significantly longer in the thigh muscle than in the other organs (T_sat_, **P = 0.004 by Kruskal–Wallis test, T_10_, T_63_, and T_90_, *P < 0.05 by Kruskal–Wallis test). The T_10_, T_63_, T_90_ and T_sa__t_ were not significantly different between brain, liver, kidney and mesentery fat. The duration of the initial plateau (T_zero_) was comparable for all organs.

The dynamic of hydrogen concentration changes before reaching saturation regardless the organ, as illustrated in Fig. 3. Measurements of saturation dynamics (T_sat_, T_90_, T_63_, T_10_, and T_zero_) are shown in Fig. 4. The T_sat_ was significantly longer and increased more gradually in muscle compared to the other examined organs (20.1 vs 6.3, 7.8, 8.2 and 9.4 min in brain, liver, kidney and mesentery fat, respectively (*P* = 0.004 in all cases), as shown in Fig. 4). Similar results were found at T_10_ (1.7 vs 0.5–0.9 min, *P* = 0.04 in all cases), T_63_ (7.4 vs 1.9–3.2 min, *P* = 0.01 in all cases), and T_90_ (14.4 vs 3.6–5.7 min, *P* = 0.01 in all cases) (Fig. 4). Changes in hydrogen concentration were similar among the brain, liver, kidney and mesentery fat, with no significant differences at T_10_, T_63_, T_90_ and T_sat_ between these organs.

The T_zero_ was comparable between muscle and the other organs (1.0 vs 0.4–0.6 min, *P* = 0.06 in all cases, Fig. 4). Similar results were found for the adjusted T_zero_ (1.2 min for muscle vs 0.4–0.8 min for other organs, *P* = 0.12 in all cases, Supplementary Table S1).

### Hydrogen desaturation dynamics

After hydrogen was inhaled, the hydrogen concentration in each organ returned to baseline (Supplementary Fig. S1). The hydrogen concentration decreased slower in muscle than in the other target organs while changes in concentration decreased with almost the same rate in brain, liver, kidney and mesentery fat.

## Discussion

In the present study, the distribution and real-time changes in hydrogen concentration in several rat organs (i.e., muscle, brain, kidney, liver, and mesentery fat) were monitored by continuous inhalation of 3% hydrogen gas. Generally, the results revealed that saturation distribution and dynamics were significantly different among the different target organs. Notably, the time needed to saturate tissues with artificially administered hydrogen and C_max_ values did not correlate with each other. To the best of our knowledge, this is the first study reporting on differences of hydrogen concentration distribution in animal tissues obtained by comparing the concentration curves.

Since molecular hydrogen can penetrate cell membranes due to its electrical neutrality and low molecular weight, many studies have suggested that the hydrogen gas can diffuse by organs immediately after supply^6–10,14,17^. A previous study conducted on rats reported that the hydrogen concentration in various organs reached the maximum levels 5 min after oral or intraperitoneal administration of a hydrogen super-rich saline solution and 1 min after intravenous injection^18^. Another study also showed that the hydrogen concentration in arterial blood and myocardium of rats similarly reached the maximum 5 min after gas inhalation^9^.

In contrast, more delayed hydrogen saturation was reported in the thigh muscle of rats^9^, suggesting that the distribution of hydrogen is not completely dependent on gaseous diffusion. In the current study it was found that T_sat_, T_90_, T_63_, and T_10_ all were significantly longer in muscle than in the brain, liver, kidney and mesentery fat. These results would be mainly explained by the fact that the blood flow in the muscle of rats is lower than that in other organs, particularly under anaesthesia^20^. Considering inhaled hydrogen would initially dissolve into the blood within the lungs, it could be distributed throughout the body by regional blood flow in addition to gaseous diffusion. Moreover, a study on the ischaemic myocardium of rats reported an increase in hydrogen concentration immediately after restoration of coronary artery blood flow^9^.

Despite the delayed saturation of hydrogen in the thigh muscle, the T_zero_ was comparable among target organs examined in the present study, even after its adjustment. This supports the speculation that the hydrogen would be supplied in large part through the blood, because the arterial blood reaches organs simultaneously regardless of flow rate. It should also be emphasized that if the hydrogen is distributed only by gaseous diffusion, changes in hydrogen concentration would depend on the distance between the face (gas supply hood was attached) and each organ, and the hydrogen would rapidly diffuse (low T_zero_) to the brain, because it is closer to the face than the thigh muscle (high T_zero_) [known as Fick’s laws]^20^.

**Figure 5:**
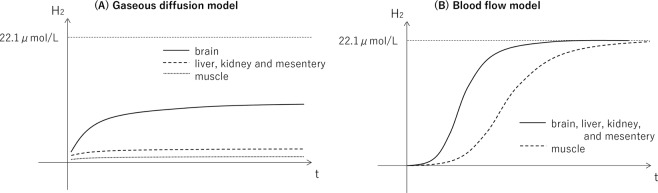
Theoretical models of hydrogen distribution. (**A**) In gaseous diffusion model, hydrogen concentration would increase more rapidly and would saturate at higher value in the brain than the muscle and other organs based on the distance between the gas supply hood (the face and head) and each organ, although maximum concentration would be lower than the administered level (22.1 μmol/L) due to high diffusion coefficient of hydrogen gas. (**B**) In blood flow model, hydrogen concentration would increase more gradually in the muscle than other organs due to lower blood flow of muscle, while maximum concentrations would be similar across organs because arterial blood would be saturated at the administered level.

Theoretical models of hydrogen distribution were descripted in Fig. 5. In gaseous diffusion model, hydrogen concentration would increase more rapidly and would saturate at higher value in the brain than the muscle and other organs based on the distance between the gas supply hood (the face and head) and each organ, although maximum concentration would be lower than the administered level (22.1 μmol/L) due to high diffusion coefficient of hydrogen gas (Fig. 5A). In blood flow model, hydrogen concentration would increase more gradually in the muscle than other organs due to lower blood flow of muscle, while maximum concentrations would be similar across organs because arterial blood would be saturated at the administered level (Fig. 5B). Comparing these theoretical models with the results of current study (Fig. 3), we believe that the hydrogen would be supplied mostly through the blood in addition to gaseous diffusion.

Although the molecular/cellular physiology underlying the differences in saturated hydrogen concentration by organs remains unclear, it has been suggested that the hydrogen accumulates in polysaccharide, such as glycogen, or in the lipid phase of cells^21,22^. The highest concentration of hydrogen in the liver observed in the present study (29.0 ± 2.6 μmol/L) was higher than the administered level (22.1 μmol/L), indicating hydrogen storage in the liver.

Contradictory results have been reported regarding the C_max_ of hydrogen. While a recent study showed that the muscle accommodated the highest concentration of hydrogen via inhalation^18^, another study reported lower concentration in the muscle than in myocardium^9^. High concentrations were also shown in the spleen and pancreas after oral or intraperitoneal administration of hydrogen^18^. On the other hand, the present study shows that the liver has the highest C_max_, whereas it considerably varies between organs.

These discrepancies are likely related to the experimental methods for measurement. For example, C_max_ can only be determined by continuous monitoring, since it is not known when the hydrogen concentration will reach a maximum in a certain organ/tissue. It is also important to underline that the hydrogen rapidly diffuses and can penetrate even glass containers^23^, therefore, it should be administered continuously and its concentration should be measured *in vivo*, not via sampled tissues, to obtain accurate saturation measurements^9^. Moreover, the administration methods should be also considered, since the hydrogen can be introduced and distributed throughout the body by both gaseous diffusion and blood flow. Accordingly, we believe that maximum concentrations obtained in the current study on continuously inhaled model are more accurate than ones in the previous studies.

The results of this study must be interpreted in the context of the study design. We only administer the 3% hydrogen with gas inhalation and its distribution to the body might differ when administered by other methods/routes or other concentrations. However, inhalation allows the application of a constant dose and by which C_max_ can be obtained appropriately. Furthermore, it was shown that the inhalation of less than 4% hydrogen is practical and safe for patients^14^. A double-blind, randomised control trials using 2% hydrogen inhalation are ongoing^11,24^. Therefore, we believe that the current results may be applicable to clinical settings.

Another limitation to our study is related to the lack of measurement of hydrogen concentration in the arterial blood, which may affect the hypotheses regarding the hydrogen distribution through the blood flow in conjunction with gaseous diffusion. Although the hydrogen in the blood has been reported in past studies^9,18^, it is technically difficult to monitor the real-time concentration as the insertion of the microsensor into major vessels causes bleeding. Loss of blood volume would significantly impair regional blood flow in organs and tissues, which could alter results considerably.

Furthermore, although negative value in hydrogen concentration is not physically correct, some negative concentrations were detected. We believe the electric signal produced by hydrogen microsensor might have been affected by temperature, salinity, or oscillation of tissue, which are known to influence electric current signals generated at anode of microsensor. However, since we performed calibration to minimize measurement error and the negative values were small and recorded only in the brain, our results would still provide the speculation that the hydrogen would be supplied mostly through the blood.

In conclusion, the present study reported on hydrogen concentrations and significantly various distributions among organs in rats exposed to continuous hydrogen gas inhalation. While thigh muscle required a longer time to saturate, the liver had the highest C_max_ with considerable variation between organs. These results are essential to elucidate the mechanisms underlying the beneficial therapeutic effects of hydrogen in mammalian systems, and future investigations using the inhalation of hydrogen gas should consider the significant differences in hydrogen distribution between organs when designing experiments and interpreting results.

## Methods

### Animals

The protocol used in the present study was approved by the Research Council and Animal Care and Use Committee of the Research Institute of Keio University (Tokyo, Japan) and performed in accordance with the guidelines for the care and use of laboratory animals established by the Japanese Pharmacological Society and the National Institutes of Health. Eight-week old male Sprague-Dawley rats (250–270 g) were purchased from Sankyo Labo Service Corporation, Inc. (Tokyo, Japan) and maintained in a temperature- and light-controlled room (20 °C, 12-h light/12-h dark cycle). The rats had free access to food and water before the experiment.

### Hydrogen gas preparation

Hydrogen gas (3%) was prepared using a hydrogen gas supply device (Nihon Kohden Co., Tokyo, Japan) and administered to rats with a rate of 0.2 L/min. Hydrogen gas stored in the device was mixed with air, then the targeted concentration was measured inside the supply device. The gas flow rate was adjusted at the output port of the device and validated with a flow metre attached to the respiratory circuit.

### Hydrogen concentration monitoring

A highly sensitive hydrogen microsensor (Unisense, Aarhus, Denmark) composed of a silicone membrane, hydrogen oxidising platinum anode, and a glass tip (diameter, 500 μm) was used to measure the hydrogen concentration in real-time. The silicone membrane at the glass tip is used for separating hydrogen from gas mixtures in tissue. The hydrogen selectively diffuses through the membrane to the hydrogen oxidizing platinum anode and the microsensor produces an electric current (signal). The electrical signal was amplified with the device’s multi meter and translated into hydrogen concentration units. Since the signal is slightly affected by temperature, salinity, or oscillation of tissue, calibration was performed using distilled water warmed to 37 °C and 3% hydrogen solution (22.1 μmol/L in water).

### Experimental protocol

Rats were anaesthetised intraperitoneally with a combination of 0.3 mg/kg medetomidine, 2.0 mg/kg midazolam, and 2.5 mg/kg butorphanol. They were appropriately anesthetized for the entire hydrogen administration procedure and until concentration levels returned to baseline. The respiratory circuit was connected to the gas supply hood, which covered the face and head of rats. Only one of the target organs was exposed at a time by minimal incision before inserting the microsensor tip to a depth of about 1 mm below the surface of the organ. The sensor body was held by a test tube holder.

To determine baseline levels, the real-time monitoring of hydrogen concentration in the target organ started before the administration of hydrogen gas, and the concentration values were recorded every 0.5 s. Inhalation of 3% hydrogen gas continued until the target tissue became saturated. At this point, the hydrogen administration was discontinued, but the target organ was monitored until the hydrogen concentration returned to baseline. Target organs included the right cerebrum, median lobe of the liver, right kidney, fat tissue of the mesentery, and muscle of the left thigh (*n* = 4–8 per organ). The hydrogen gas microsensor was calibrated after each measurement.

### Statistical analysis

Descriptive statistics are presented as the mean ± standard deviation or as a percentage. Inter-organ comparisons of the concentration curves were performed using analysis of variance and/or Kruskal–Wallis, unpaired *t-*, Mann–Whitney *U*-, Chi-square or Fisher’s exact tests when appropriate. A two-sided *α* threshold of 0.05 was considered statistically significant for all hypotheses tested. All statistical analyses were conducted using SPSS ver.24 software (SPSS, Chicago, IL, USA) and Microsoft Excel (Microsoft, Redmond, WA, USA).

## Supplementary information

Supplementary Figure and Table

Supplementary data File

## Data Availability

All data generated or analysed during this study are included in this published article and its Supplementary Information files.
