# Supplementary material for: Hydrogen gas distribution in organs after inhalation: Real-time monitoring of tissue hydrogen concentration in rat
Source: Sci Rep. 2019 Feb 4;9:1255. doi: 10.1038/s41598-018-38180-4 (PMC6362202; doi:10.1038/s41598-018-38180-4)
Supplement: Supplementary file 1 — Supplementary Figure and Table [file 41598_2018_38180_MOESM1_ESM.docx]

# Hydrogen gas distribution in organs after inhalation: Real-time monitoring of tissue hydrogen concentration in rat

**Ryo Yamamoto, MD,^1^ Koichiro Homma, MD, PhD,^1,*^ Sayuri Suzuki, PhD,^1^ Motoaki Sano, MD, PhD, ^2^ Junichi Sasaki, MD, PhD^1^**

^1^Department of Emergency and Critical Care Medicine, Keio University School of Medicine, Tokyo, Japan

^2^Department of Cardiology, Keio University School of Medicine, Tokyo, Japan

*Corresponding author:

Koichiro Homma, MD, PhD, Department of Emergency and Critical Care Medicine, Keio University School of Medicine, Tokyo, Japan

Email: homma@keio.jp

**Supplementary figure S1**

**
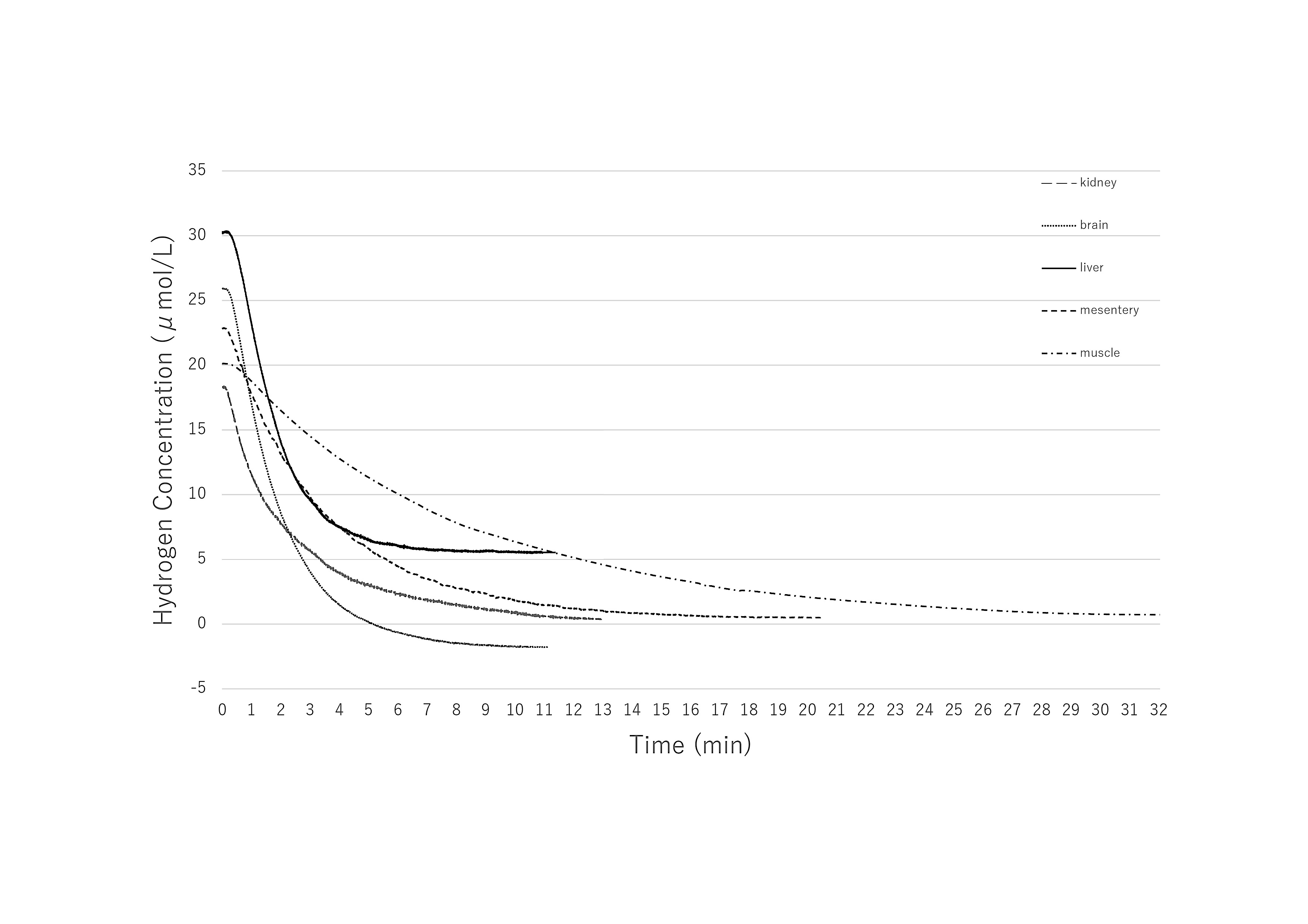
**

**Supplementary figure legend**

**Supplementary Figure S1. Hydrogen desaturation dynamics.** After terminating inhalation, the hydrogen concentration in each organ returned to baseline. The hydrogen concentration decreased more gradually in thigh muscle versus the other organs, while the concentration decreased at similar rates in the brain, liver, kidney and mesentery fat.

**Supplementary Table**

**Table S1. Time measurements in organs**

|  | Time measurements (min) | | |  |  |  |
| --- | --- | --- | --- | --- | --- | --- |
|  | Tzero | T10 | T63 | T90 | Tsat | Tzero-adjusted |
| Brain | 0.36 | 0.52 | 1.89 | 3.62 | 6.31 | 0.43 |
| Liver | 0.48 | 0.66 | 2.07 | 4.21 | 7.82 | 0.46 |
| Kidney | 0.59 | 0.74 | 2.12 | 4.56 | 8.22 | 0.54 |
| Mesentery | 0.61 | 0.89 | 3.17 | 5.73 | 9.36 | 0.79 |
| Muscle | 1.01 | 1.66 | 7.44 | 14.43 | 20.15 | 1.16 |
